# The Pathogens Spillover and Incidence Correlation in Bumblebees and Honeybees in Slovenia

**DOI:** 10.3390/pathogens10070884

**Published:** 2021-07-12

**Authors:** Metka Pislak Ocepek, Ivan Toplak, Urška Zajc, Danilo Bevk

**Affiliations:** 1 Institute of Pathology, Wild Animals, Fish and Bees, Veterinary Faculty, University of Ljubljana, Gerbičeva 60, 1000 Ljubljana, Slovenia; 2 Virology Unit, Institute of Microbiology and Parasitology, Veterinary Faculty, University of Ljubljana, Gerbičeva 60, 1000 Ljubljana, Slovenia; ivan.toplak@vf.uni-lj.si; 3 Bacteriology Unit, Institute of Microbiology and Parasitology, Veterinary Faculty, University of Ljubljana, Gerbičeva 60, 1000 Ljubljana, Slovenia; urska.zajc@vf.uni-lj.si; 4 Department of Organisms and Ecosystems Research, National Institute of Biology, Večna pot 111, 1000 Ljubljana, Slovenia; danilo.bevk@nib.si

**Keywords:** bumblebees, honeybees, viruses, *Nosema* spp., *Crithidia bombi*, *Apicystis bombi*, *Lotmaria passim*, pathogens transmission

## Abstract

Slovenia has a long tradition of beekeeping and a high density of honeybee colonies, but less is known about bumblebees and their pathogens. Therefore, a study was conducted to define the incidence and prevalence of pathogens in bumblebees and to determine whether there are links between infections in bumblebees and honeybees. In 2017 and 2018, clinically healthy workers of bumblebees (*Bombus* spp.) and honeybees (*Apis mellifera*) were collected on flowers at four different locations in Slovenia. In addition, bumblebee queens were also collected in 2018. Several pathogens were detected in the bumblebee workers using PCR and RT-PCR methods: 8.8% on acute bee paralysis virus (ABPV), 58.5% on black queen cell virus (BQCV), 6.8% on deformed wing virus (DWV), 24.5% on sacbrood bee virus (SBV), 15.6% on Lake Sinai virus (LSV), 16.3% on *Nosema bombi*, 8.2% on *Nosema ceranae*, 15.0% on *Apicystis bombi* and 17.0% on *Crithidia bombi*. In bumblebee queens, only the presence of BQCV, *A. bombi* and *C. bombi* was detected with 73.3, 26.3 and 33.3% positive samples, respectively. This study confirmed that several pathogens are regularly detected in both bumblebees and honeybees. Further studies on the pathogen transmission routes are required.

## 1. Introduction

Honeybees and wild pollinators play an essential role in plant pollination, which is important for both agricultural production and biodiversity conservation [1,2]. In addition to the honeybees, the role of wild pollinators is also very important, as they are in many cases even more effective than honeybees and it is now known that honeybees can complement but not replace wild pollinators [3]. Evidence of pollinator decline and disappearance is alarming in many countries around the world [4]. However, the importance of bumblebees has only been increasingly researched in recent years, when the proportion of publications on bumblebee conservation began to grow exponentially [5]. In Europe, more than 20% of bumblebees are threatened with extinction and populations are declining in nearly 50% of species [4]. Important reasons for the decline of pollinator populations and diversity are not only habitat degradation and loss mainly due to urbanisation [6], intensive agriculture, which also involves the use of pesticides [7,8] and climate changes [9,10], but also various pathogens that affect wild pollinators [5,11,12,13,14].

Many diseases occur in both honeybees and wild bees, but less is known about pathogen transmission routes between them [12,15,16,17]. The collection of nectar and pollen by pollinators on flowers allows transmission of pathogens between different pollinator species [18,19,20,21]. Typically, pathogen transmission occurs from farmed species, such as honeybees and commercial bumblebee farms, to wild species [22,23,24,25]. Two main mechanisms of parasite spread between managed and wild populations are spillover and spillback. Facilitation also leads to a decline in wild bees, while a high density of managed bees leads to wild bees being stressed and more susceptible to infection [24], which is also influenced by adequate food in the environment [26].

The commonly found pathogens in bumblebees are *Apicystis bombi* (*A. bombi*), *Crithidia bombi* (*C. bombi*), *Nosema bombi* (*N. bombi*), *Nosema ceranae* (*N. ceranae*) and several viruses such a Deformed wing virus (DWV), Acute bee paralysis virus (ABPV), Black queen cell virus (BQCV), Sacbrood bee virus (SBV) and Lake Sinai virus (LSV) [11,12,27]. Many of these pathogens primarily or commonly infect honeybees. Infection with *C. bombi*, an intestinal trypanosome, can cause behavioural changes and disturbances in flower colour perception in bumblebees, reducing their ability to forage [28]. *C. bombi* infection can also reduce the survival of bumblebee queens in hibernation [29] and their ability to successfully establish colonies [30]. *Lotmaria passim* (*L. passim*) is a highly prevalent trypanosomatid in honeybees but its pathogenicity/impact on the bumblebee health is not yet clear [31,32]. *Nosema* spp. is a microsporidian that is widely distributed among honeybees and bumblebees. *N. bombi* has been shown to have negative effects on the vitality of bumblebees by impairing the ability of queens to form new colonies, affecting colony size and the vitality of young queens and drones and shortening the life span of workers and drones [33,34,35]. *N. ceranae* is primarily a pathogen of the Asian bee *Apis ceranae* and is also found in bumblebee colonies worldwide. As previously confirmed, *N. ceranae* can infect bumblebees, affecting their longevity [12,36], but this has not always been demonstrated [37]. *A. bombi* is a neogregarine pathogen that is recognised as one of the causes of bumblebee declines [38]. Experimentally, high virulence on bumblebee queens has been found in individuals with *A. bombi* infection [39]. Spillover from farmed bumblebees to wild bumblebees is suspected due to higher pathogens prevalence around greenhouses with commercial bumblebees [40].

Several viruses infect honeybees and can cause pathological changes at different stages. The use of molecular methods in recent studies has confirmed the occurrence of these honeybee viruses in commercial and wild bumblebees as well [12,14,27]. In bumblebees, virus replication with some clinical changes has also been recognised [41,42]. Clinical signs of DWV infection have been found in *B. terrestris* and *B. pascuorum* [43] and an association between wing deformities and virus localisation in the bumblebee head has been confirmed in commercially bred colonies of *B. terrestris* [44]. By sequencing and phylogenetic analysis of individual viruses, the same strains of ABPV, BQCV, SBV and LSV were identified in bumblebees and honeybees, confirming the assumption that viruses are successfully transmitted between different species [27]. Very little is known about the impact of bee viruses on bumblebee decline and the occurrence of other, unexplored viruses in bumblebees [45].

Over 500 different species of wild bees have been found in Slovenia, of which 35 are bumblebees (*Bombus* spp.). The trend that wild bee populations are declining has also been observed in Slovenia [46]. It is extremely important to identify all threatening factors in order to determine the appropriate protection measures. On the other hand, Slovenia is a country with a long beekeeping tradition and is home to the Carniolan honeybee (*Apis mellifera carnica*), which is protected by law. Slovenia is only 20,271 square kilometres in size and has a population of about 2 million, but according to the national register of apiaries there are about 11,000 beekeepers with more than 200,000 honeybee colonies and the number of honeybee colonies in Slovenia has grown rapidly in recent years (data from Ministry of agriculture, forestry and food for year 2020). There is a lack of knowledge about the presence of some pathogens in wild pollinators in Slovenia and about the possible effects of high honeybee density on wild pollinators.

The aim of this research is to monitor the health status of the bumblebee population and to compare the prevalence of pathogens in bumblebees with the status of honeybees at four different locations in Slovenia. We were also interested in how bumblebees become infected, whether with transmission from queen to nest, or whether bumblebee workers become infected when collecting food on flowers. Therefore, the study of prevalence of some pathogens on bumblebees compared to honeybees was conducted in Slovenia in 2017 and 2018.

## 2. Results

A total of 147 bumblebees and 8 pooled samples of honeybees from four different locations in Slovenia and 15 bumblebee queens from two locations were analysed. Overall, 8.8% of bumblebee workers were detected positive on ABPV, 58.5% were positive on BQCV, 6.8% were positive on DWV, 24.5% were positive on SBV, 15.6% were positive on LSV, 16.3% were positive on *N. bombi*, 8.2% were positive on *N. ceranae*, 15.0% were positive on *A. bombi* and 17.0% were positive on *C. bombi* (Table 1). For honeybee samples, 62.5% were positive on ABPV, 100% were positive on BQCV, 12.5% were positive on chronic bee paralysis virus (CBPV), 25% were positive on DWV, 50% were positive on SBV, 87.5% were positive on LSV, 87.5% were positive on *N. ceranae*, 12.5% were positive on *A. bombi*, 75.0% were positive on *C. bombi* and 100% were positive on *L. passim*. There were no positive honeybee samples on *N. bombi* and *N. apis* (Table 2). In bumblebee queens, the presence of BQCV, *A. bombi* and *C. bombi* was detected with 73.3, 26.7 and 33.3% positive bumblebee queens, respectively. There were no bumblebee queens positive on CBPV, *N. apis* and *L. passim* (Table 3).

**Figure 1:**
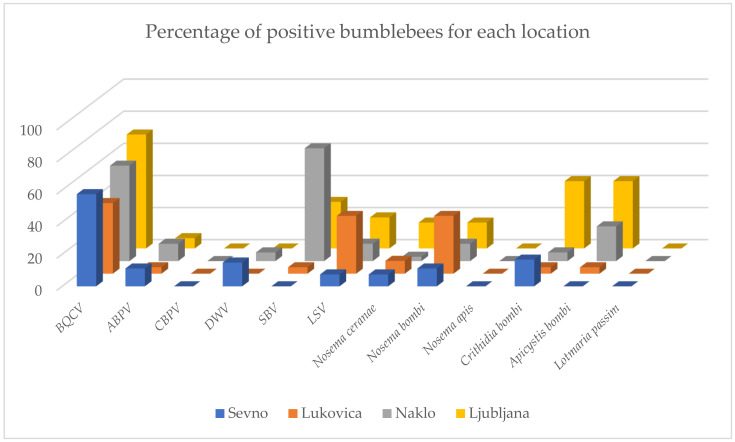
Comparison of positive bumblebee samples at four locations (Sevno, Lukovica, Naklo and Ljubljana). Results are presented together as a percentage of positive samples for samplings in 2017 and 2018 for all tested pathogens (BQCV = black queen cell virus, ABPV = acute bee paralysis virus, CBPV = chronic bee paralysis virus, DWV = deformed wing virus, SBV = sacbrood bee virus, LSV = Lake Sinai virus).

**Figure 2:**
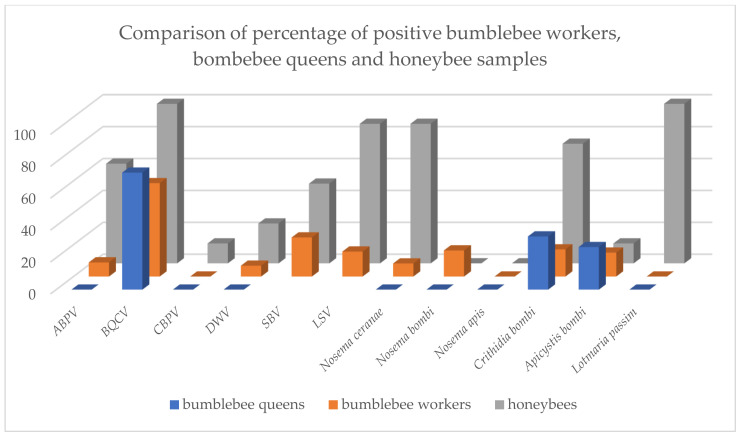
Comparison of percentage of positive bumblebee worker, bumblebee queen and honeybee samples. Results for both years of sampling and for all four locations are presented together for all tested pathogens (BQCV = black queen cell virus, ABPV = acute bee paralysis virus, CBPV = chronic bee paralysis virus, DWV = deformed wing virus, SBV = sacbrood bee virus, LSV = Lake Sinai virus).

Results for bumblebee workers were presented together for samplings in 2017 and 2018 and calculated as a percentage of total positive samples for each location (Figure 1). To compare the results for different species, results for honeybees were also summed for 2017 and 2018 and calculated as a percentage of positive samples. Results for bumblebee queens sampled in 2018 were calculated as a percentage of positive samples. Calculated honeybee and bumblebee queen results were compared to bumblebee worker results (Figure 2).

**Figure 3:**
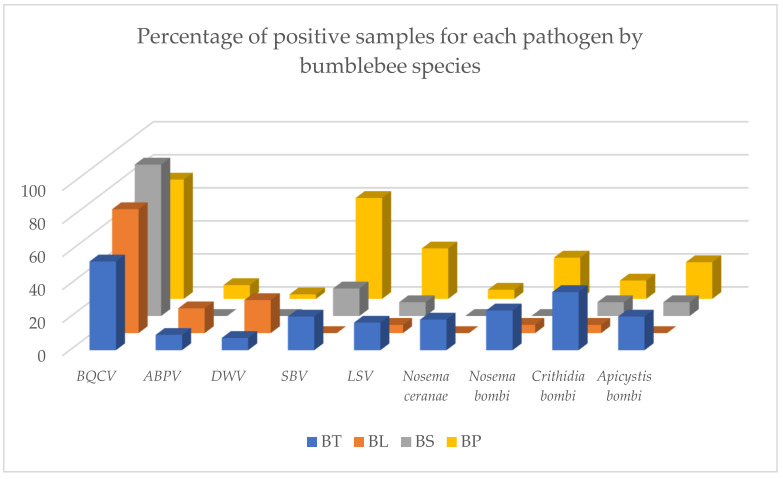
Comparison of percentage of positive bumblebee worker samples for each pathogen (BQCV= black queen cell virus, ABPV= acute bee paralysis virus, DWV= deformed wing virus, SBV= sacbrood bee virus, LSV= Lake Sinai virus), analysed by bumblebee species (BT = *Bombus terrestris/lucorum*, BL = *Bombus lapidarious*, BS = *Bombus sylvarum*, BP= *Bombus pascuorum*).

The results of determining the presence of pathogens in worker bumblebees were also analysed according to the bumblebee species. Since only three specimens of *B. hortorum* and two of *B. humilis* were collected in this study; these two species were excluded from the analysis. The results of analysis are shown in Figure 3 as percentage of positive samples for each pathogen. As no positive samples were determined for CBPV, *N. apis* and *L. passim*, these pathogens are not included.

**Figure 4:**
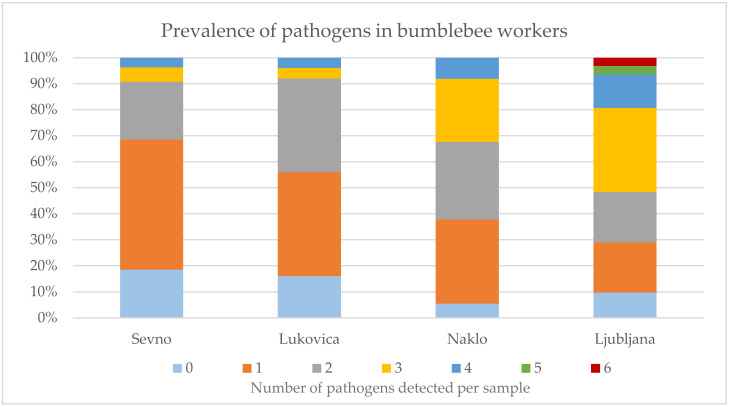
Results of pathogen prevalence analysis for each bumblebee worker. Numbers 0–6 represent the number of pathogens detected per sample; results are presented as percentage of samples with number of pathogens detected.

For all sampled bumblebee workers, the analysis of the number of individual pathogens confirmed in each bumblebee was done. At the Sevno location, no pathogen was detected in 10 (18.5%) samples, while one, two, three and four pathogens were detected in 27 (50%), 12 (22.2%), 3 (5.6%) and 2 (3.7%) samples, respectively. At Lukovica location, 4 (16%) of the bumblebee workers were without pathogens, while one, two, three and four pathogens were detected in 10 (40%), 9 (36%), 1 (4%) and 1 (4%) sample, respectively. At Naklo location no pathogen was detected in 2 (5.4%) samples, one, two, three and four pathogens were detected in 12 (32.4%), 11 (29.7%), 9 (24.3%) and 3 (8.1%) samples, respectively. At Ljubljana location, no pathogen was detected in 3 (9.7%) samples, one, two, three and four pathogens were detected in 6 (19.4%), 6 (19.4%), 10 (32.3%) and 4 (12.9%) samples, respectively, while at this location 1 (3.2%) bumblebee worker was infected with five pathogens and 1 (3.2%) with six pathogens (Figure 4). All four bumblebee sampling sites are in the same category in terms of the honeybee colonies density of 11.6–13.5 honeybee colonies per km^2^ (data from national register of apiaries for year 2020, Ministry of agriculture, forestry and food).

## 3. Discussion

This study is the first comprehensive investigation of the occurrence and prevalence of various pathogens in bumblebees in Slovenia. Our results also show that bumblebees can be simultaneously infected with several pathogens and that many of them are shared with honeybees. In bumblebees, ABPV, BQCV, DWV, SBV and LSV were confirmed, while no CBPV was detected in healthy bumblebees, as expected, since CBPV is one of the most pathogenic viruses of adult honeybees [47]. Among the detected viruses, most of the bumblebees were positive for BQCV (58.5%), followed by SBV (24.5%), LSV (15.6%), ABPV (8.8%) and DWV (6.8%). The collected 10 honeybees at each of the four locations were pooled into one pool sample per sampling day, thus eight honeybee samples were included in this study to prove the presence or absence of the individual pathogen at the time of collection also among the honeybees. Although precise data on the proportion of positive individual honeybees were not obtained, the results of these samples serve as good evidence that the detected pathogen was present locally at the time of samplings and a comparison with prevalence in bumblebees was possible. Among the honeybee samples, BQCV (100%) was the most frequently detected virus, followed by LSV (87.5%), ABPV (62.5%), SBV (50.0%), DWV (25.0%) and CBPV. (12.5%). When comparing these results with previously published data [48], the most frequently detected virus in honeybees in Slovenia was also BQCV (83.3%), followed by DWV (70%), ABPV (40%), CBPV (18.3%) and SBV (8.3%). In data interpretation, it should be noted that this time we collected clinically healthy specimens of honeybees on flowers, whereas in a previous study we collected samples from honeybee colonies with some notable pathology, and this is the main reason for the observed differences in prevalence. The results of this study showed that CBPV was not detected in any of the bumblebee samples, although this virus is present and regularly/yearly detected in Slovenia mainly in clinically diseased honeybees. The same observation for CBPV was also reported by some other authors in their previous studies [11,13,14].

In addition to viruses, *N. ceranae*, *N. bombi*, *C. bombi* and *A. bombi* have also been detected in bumblebee workers, confirming the observation of previous studies [11,29,38]. The highest prevalence was found for *C. bombi* (17%), followed by *N. bombi* (16.3%), *A. bombi* (15%) and *N. ceranae* (8.2%), while we could not confirm *N. apis* and *L. passim*. In our experience, *L. passim* is very common in honeybees in Slovenia, as well as in some other countries [31,32]. Therefore, we included it in our study to see if it is also transmitted to bumblebees, but we could not detect it in any bumblebee sample, although all collected honeybee samples at the same locations were positive. It could be concluded that bumblebees are not susceptible to infection with *L. passim* and that various honeybee pathogens are not present in bumblebees only as a result of contamination on flowers. In Slovenia, *N. apis* was not detected in honeybee samples for years, so it was not surprising that all bumblebee samples were negative. *N. ceranae*, on the other hand, is frequently diagnosed in honeybees in Slovenia, which is also evident in this study (7 out of 8 pool samples were detected positive). Our results show that *N. ceranae* also infects bumblebees. Despite the fact, that *N. bombi*, *C. bombi* and *A. bombi* are pathogens, known to infect bumblebees [11,28,29,38], we found *C. bombi* and *A. bombi* also in honeybees.

Comparing the data presented for bumblebee workers, bumblebee queens and honeybee samples, a correlation of the occurrence and prevalence of pathogens between honeybees and bumblebees can already be evident for individual pathogens (Figure 2). According to these results and studies by other authors [11,12,13,14,24,25,27] there is spillover of pathogens between managed honeybees and wild bumblebees. We found the bumblebee pathogens *C. bombi* (6 positive pool samples out of 8) and *A. bombi* (1 positive pool sample out of 8) also in honeybees. This suggests that the spillback effect is probably also present, mainly due to the high density of honeybee colonies/apiaries in Slovenia, which was more than 10 colonies per km^2^ in 2020, according to the national register of apiaries.

The results are also analysed by bumblebee species. In the Figure 3 same differences between species are evident, but we cannot say that there is one species of bumblebee that is more or less healthy, since there are several pathogens present in each species. The different number of samples for each species must be taken into account. Since we had only three and two samples of *B. hortorum* and *B. humilis*, respectively, these two species were excluded from the analysis. It is obvious, that in species with a higher number of examined samples (*B. terrestis/lucorum* and *B. pascuorum*) more pathogens were detected, as the probability of collecting infected specimen is higher. Despite a few differences between species, there are no significant results, except for a slightly higher percentage of positive *B. pascuorum* samples in the SBV, for which we do not know the reason. However, it may be useful to examine *B. pascuorum* nests for the presence of clinical signs of SBV in the future.

**Figure 5:**
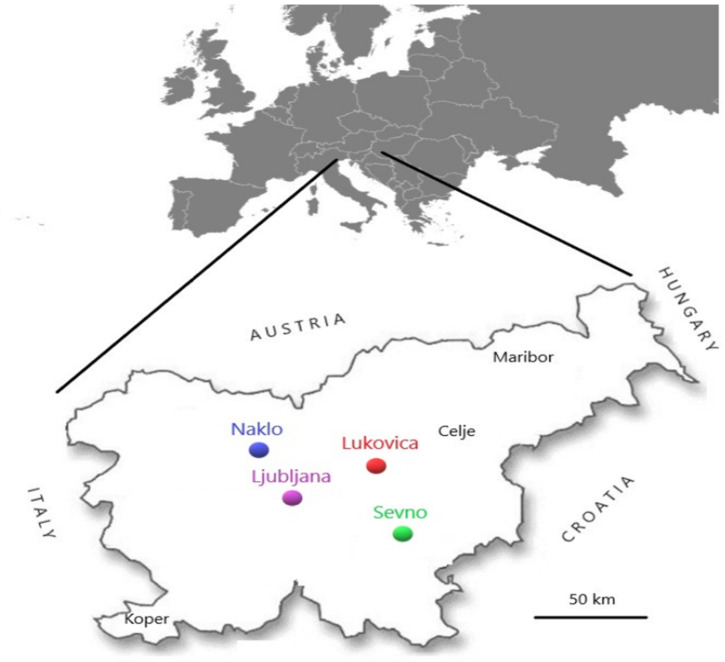
Four locations (Sevno, Lukovica, Naklo and Ljubljana) on the map of Slovenia where samples were collected in 2017 and 2018.

To monitor the health status of the bumblebee population in Slovenia, the results were analysed and interpreted according to the number of pathogens detected in each bumblebee. Between zero and four pathogens were detected in most of bumblebee samples, while at the location of Ljubljana one bumblebee was identified with five pathogens and another with six pathogens. The presented data on prevalence analysis for each bumblebee worker sample (Figure 5) showed important differences between four locations, the least pathogens in individual bumblebees were found at the location Sevno, followed by Lukovica, Naklo and the most at the location Ljubljana. We do not know the real reason for this result, as the locations do not differ significantly in terms of honeybee colony density, perhaps the proximity of urban area (Ljubljana is the capital of Slovenia) is more stressful for bumblebees.

In our previously published research study based on a molecular epidemiological approach and phylogenetic comparison of detected ABPV, BQCV, SBV and LSV in different species, we confirmed that several viruses are undoubtedly transmitted between bees and bumblebees [27]. To identify the possible ways of transmitting pathogens between different species, 15 bumblebee queens were sampled in April of the second year of the study and included in the comparison. Since bumblebee queens are the only ones that overwinter and form a new colony during the season, these samples were tested for ABPV, BQCV, CBPV, DWV, *N. bombi*, *N. ceranae*, N. *apis*, *A. bombi*, *C. bombi* and *L. passim* for the first time. Only the presence of BQCV, *A. bombi* and *C. bombi* was detected in queens. Regarding this observation, it seems that *Nosema* spp. and some viruses are not transmitted by queens, indicating the possibility of infection of bumblebee workers by indirect contacts on flowers. If pathogens are transmitted between different species in this way, they may also be transmitted among honeybees, especially if there is a high density of honeybee colonies in areas with a rich honey flow. When diseases spread among managed bees, pathogens multiply more easily and are transmitted even more to wild pollinators [25]. This also adds a new dimension to the health of honeybees and other pollinators that should be considered by beekeepers and policy-makers. Even more, effects in the nature are so closely related that we cannot separate the care of honeybees from the conservation of wild pollinators. However, further studies are needed to confirm the possibility of disease transmission between honeybees and various pollinators during their pollination activities.

## 4. Materials and Methods

In August 2017 and August 2018, a total of 147 clinically healthy bumblebee workers of different species: *Bombus terrestis/ lucorum*, *B. lapidarious*, *B. sylvarum*, *B. pascuorum*, *B. hortorum* and *B. humilis* (Table 4) were individually collected on flowers in nature. Sampling was carried out at four different locations in Slovenia (Figure 5): 24 and 30 bumblebees were collected in Sevno in 2017 and 2018, 10 and 15 in Lukovica, 20 and 17 in Naklo and 11 and 20 in Ljubljana, respectively. At the same locations (Sevno, Lukovica, Naklo and Ljubljana) on the day of bumblebee sampling, also 10 clinically healthy honeybee workers (*Apis mellifera carnica*) were collected on flowers, for a total of 80 clinically healthy honeybee workers. In April 2018, also 15 clinically healthy bumblebee queens were collected on the same way as bumblebee workers on flowers, 10 samples at the Sevno site (5 samples of *B. terrestris/lucorum* and 5 samples of *B. lapidarious*) and 5 samples of *B. pascuorum* at the Ljubljana site. All samples were frozen and stored at minus 60 °C until use.

In the laboratory, each bumblebee was placed in an Ultra-Turrax DT-20 tube (IKA, Germany) and 3 mL of RPMI 1640 medium was added. Clinically healthy honeybees collected on the same day at each location were pooled (10 bees from the same location and at the same time of sampling in one pool) and in laboratory 5 mL of RPMI 1640 medium (Gibco, UK) was added to each sample. The samples were homogenised, and 1 mL of the suspension was taken for isolation of DNA before centrifugation. The remainder was centrifuged at 2500× *g* for 5 min. Total RNA was isolated from each sample using the QIAamp viral RNA mini kit (Qiagen, Germany) according to the manufacturer’s instructions.

DNA was isolated using a commercial isolation kit (Institute of Metagenomics and Microbial Technologies-IMMT, Slovenia). Briefly, 1 mL of the mixture was added to a 2-mL tube containing ≤106-μm-diameter glass beads (Sigma-Aldrich, St. Louis, MI, USA) and centrifuged at 10,000× *g* for 5 min. The pellet was resuspended in 392 µL of lysis buffer and 8 µL of proteinase K (Sigma-Aldrich, St. Louis, MI, USA). This was followed by bead beating on a MagNALyser device (Roche, Basel, Switzerland), at 6400 rpm for 60 s and incubation at 56 °C for 15 min. Bead beating and incubation were repeated three times and twice, respectively. The rest of the isolation was performed according to the manufacturer’s protocol.

The RNA of six honeybee viruses in bumblebee workers: ABPV, BQCV, CBPV, DWV, SBV and LSV was detected by specific reverse transcription and polymerase chain reaction method (RT-PCR) as previously described [27,48]. Results were considered positive based on the size of the RT-PCR products in the agarose gel when the expected product size was present (ABPV 452 nt, BQCV 770 nt, CBPV 570 nt, DWV 504 nt, SBV 814 nt and LSV 603 nt). Isolated RNA from bumblebee queens was tested for ABPV, BQCV, CBPV and DWV as described above.

Isolated DNA from each bumblebee worker and queen was used to detect *N. bombi*, *N. ceranae*, *N. apis*, *A.s bombi*, *C. bombi* and *L. passim*. Polymerase chain reactions (PCR) and real-time polymerase chain reaction (qPCR) were performed according to previously published protocols [49,50,51].

## 5. Conclusions

In 147 bumblebees tested, the prevalence of ABPV, BQCV, DWV, SBV, LSV and *N. ceranae*, *N. bombi*, *C. bombi* and *A. bombi* was detected for the first time in Slovenia, while honeybees sampled at the same time and locations were positive for ABPV, BQCV, CBPV, DWV, SBV, LSV, *N. ceranae*, *C. bombi*, *A. bombi* and *L. passim*. In bumblebee queens, only BQCV, *C. bombi* and *A. bombi* were diagnosed.

The study raised some new questions regarding the transmission of pathogens between honeybees and bumblebees. However, it must be kept in mind that many factors can have an impact on surviving pollinators, including the transmission of pathogens from managed bees to wild pollinators. The evident spillover is why we need to put more attention also in good care of managed bees in order to preserve wild bees.

## Figures and Tables

**Table 1:** Results of laboratory tests, obtained by RT-PCR and PCR methods of bumblebee worker samples at four locations (Sevno, Lukovica, Naklo and Ljubljana), in two years (2017, 2018) and for different bumblebee species (BT = *Bombus terrestris/lucorum*, BL = *Bombus lapidarious*, BS = *Bombus sylvarum*, BP = *Bombus pascuorum*, BHO = *Bombus hortorum*, BHU = *Bombus humilis*). Results are presented as number of positive samples/number of tested samples and % of positive samples (in bracket) for each pathogen, year, bumblebee species and location of sampling.

		ABPV	BQCV	CBPV	DWV	SBV	LSV	*Nosema ceranae*	*Nosema bombi*	*Nosema apis*	*Crithidia bombi*	*Apicystis bombi*	*Lotmaria passim*
Sevno 2017	BT	1/9 (11.1%)	0/9 (0%)	0/9 (0%)	4/9 (44.4%)	0/9 (0%)	0/9 (0%)	0/9 (0%)	1/9 (11.1%)	0/9 (0%)	1/9 (11.1%)	0/9 (0%)	0/9 (0%)
	BL	3/10 (30%)	6/10 (60%)	0/10 (0%)	4/10 (40%)	0/10 (0%)	0/10 (0%)	0/10 (0%)	0/10 (0%)	0/10 (0%)	0/10 (0%)	0/10 (0%)	0/10 (0%)
	BP	2/5 (40%)	0/5 (0%)	0/5 (0%)	0/5 (0%)	0/5 (0%)	0/5 (0%)	0/5 (0%)	0/5 (0%)	0/5 (0%)	0/5 (0%)	0/5 (0%)	0/5 (0%)
Sevno 2018	BT	0/10 (0%)	7/10 (70%)	0/10 (0%)	0/10 (0%)	0/10 (0%)	2/10 (20%)	4/10 (40%)	4/10 (40%)	0/10 (0%)	7/10 (70%)	0/10 (0%)	0/10 (0%)
	BL	0/10 (0%)	9/10 (90%)	0/10 (0%)	0/10 (0%)	0/10 (0%)	1/10 (10%)	0/10 (0%)	1/10 (10%)	0/10 (0%)	1/10 (10%)	0/10 (0%)	0/10 (0%)
	BS	0/10 (0%)	9/10 (90%)	0/10 (0%)	0/10 (0%)	0/10 (0%)	1/10 (10%)	0/10 (0%)	0/10 (0%)	0/10 (0%)	0/10 (0%)	0/10 (0%)	0/10 (0%)
Lukovica 2017	BT	1/5 (20%)	0/5 (0%)	0/5 (0%)	0/5 (0%)	1/5 (20%)	0/5 (0%)	0/5 (0%)	1/5 (20%)	0/5 (0%)	1/5 (20%)	1/5 (20%)	0/5 (0%)
	BP	0/5 (0%)	0/5 (0%)	0/5 (0%)	0/5 (0%)	0/5 (0%)	2/5 (40%)	0/5 (0%)	0/5 (0%)	0/5 (0%)	0/5 (0%)	0/5 (0%)	0/5 (0%)
Lukovica 2018	BT	0/5 (0%)	5/5 (100%)	0/5 (0%)	0/5 (0%)	0/5 (0%)	2/5 (40%)	1/5 (20%)	0/5 (0%)	0/5 (0%)	0/5 (0%)	0/5 (0%)	0/5 (0%)
	BP	0/10 (0%)	6/10 (60%)	0/10 (0%)	0/10 (0%)	0/10 (0%)	5/10 (50%)	1/10 (10%)	8/10 (80%)	0/10 (0%)	0/10 (0%)	0/10 (0%)	0/10 (0%)
Naklo 2017	BT	2/10 (20%)	6/10 (60%)	0/10 (0%)	0/10 (0%)	8/10 (80%)	2/10 (20%)	1/10 (10%)	2/10 (20%)	0/10 (0%)	0/10 (0%)	2/10 (20%)	0/10 (0%)
	BP	0/10 (0%)	2/10 (20%)	0/10 (0%)	1/10 (10%)	8/10 (80%)	0/10 (0%)	0/10 (0%)	0/10 (0%)	0/10 (0%)	1/10 (10%)	1/10 (10%)	0/10 (0%)
Naklo 2018	BS	0/2 (0%)	2/2 (100%)	0/2 (0%)	0/2 (0%)	2/2 (100%)	0/2 (0%)	0/2 (0%)	0/2 (0%)	0/2 (0%)	1/2 (50%)	1/2 (50%)	0/2 (0%)
	BP	0/10 (0%)	7/10 (70%)	0/10 (0%)	0/10 (0%)	7/10 (70%)	1/10 (10%)	0/10 (0%)	1/10 (10%)	0/10 (0%)	0/10 (0%)	2/10 (20%)	0/10 (0%)
	BHO	2/3 (66.7%)	3/3 (100%)	0/3 (0%)	0/3 (0%)	1/3 (33.3%)	1/3 (33.3%)	0/3 (0%)	1/3 (33.3%)	0/3 (0%)	0/3 (0%)	1/3 (33.3%)	0/3 (0%)
	BHU	0/2 (0%)	2/2 (100%)	0/2 (0%)	1/2 (50%)	0/2 (0%)	0/2 (0%)	0/2 (0%)	0/2 (0%)	0/2 (0%)	0/2 (0%)	1/2 (50%)	0/2 (0%)
Ljubljana 2017	BT	0/5 (0%)	2/5 (40%)	0/5 (0%)	0/5 (0%)	1/5 (20%)	1/5 (20%)	0/5 (0%)	0/5 (0%)	0/5 (0%)	1/5 (20%)	0/5 (0%)	0/5 (0%)
	BP	0/6 (0%)	4/6 (66.7%)	0/6 (0%)	0/6 (0%)	0/6 (0%)	1/6 (16.7%)	1/6 (16.7%)	0/6 (0%)	0/6 (0%)	1/6 (16.7%)	1/6 (16.7%)	0/6 (0%)
Ljubljana 2018	BT	1/10 (10%)	9/10 (90%)	0/10 (0%)	0/10 (0%)	1/10 (10%)	2/10 (20%)	4/10 (40%)	5/10 (50%)	0/10 (0%)	9/10 (90%)	8/10 (80%)	0/10 (0%)
	BP	1/10 (10%)	7/10 (70%)	0/10 (0%)	0/10 (0%)	7/10 (70%)	2/10 (20%)	0/10 (0%)	0/10 (0%)	0/10 (0%)	2/10 (20%)	4/10 (40%)	0/10 (0%)

**Table 2:** Results of laboratory tests of honeybee worker samples collected in 2017 and 2018 at four locations (Sevno, Lukovica, Naklo and Ljubljana). Results obtained by RT-PCR and PCR methods are presented as positive (+) or negative (−) sample for each pathogen tested.

Location and Year of Sampling	ABPV	BQCV	CBPV	DWV	SBV	LSV	*Nosema ceranae*	*Nosema bombi*	*Nosema apis*	*Crithidia bombi*	*Apicystis bombi*	*Lotmaria passim*
Sevno 2017	+	+	−	−	−	−	+	−	−	−	−	+
Sevno 2018	−	+	−	−	−	+	+	−	−	+	−	+
Lukovica 2017	−	+	−	−	−	+	+	−	−	+	−	+
Lukovica 2018	−	+	−	−	−	+	+	−	−	−	−	+
Naklo 2017	+	+	−	−	+	+	−	−	−	+	+	+
Naklo 2018	+	+	+	+	+	+	+	−	−	+	−	+
Ljubljana 2017	+	+	−	+	+	+	+	−	−	+	−	+
Ljubljana 2018	+	+	−	−	+	+	+	−	−	+	−	+

**Table 3:** Results of laboratory testing obtained by RT-PCR and PCR methods of 15 bumblebee queens of three species (BT = *Bombus terrestris/lucorum*, BL = *Bombus lapidarious*, BP = *Bombus pascuorum*), collected in 2018 from two locations (Sevno n = 10 samples and Ljubljana n = 5 samples). Results are presented as number of positive queen samples/ number of tested samples and % of positive samples (in bracket) for each pathogen, bumblebee species and location of sampling.

		ABPV	BQCV	CBPV	DWV	*Nosema ceranae*	*Nosema bombi*	*Nosema apis*	*Crithidia bombi*	*Apicystis bombi*	*Lotmaria passim*
Sevno	BT	0/5 (0%)	5/5 (100%)	0/5 (0%)	0/5 (0%)	0/5 (0%)	0/5 (0%)	0/5 (0%)	2/5 (40%)	2/5 (40%)	0/5 (0%)
	BL	0/5 (0%)	3/5 (60%)	0/5 (0%)	0/5 (0%)	0/5 (0%)	0/5 (0%)	0/5 (0%)	1/5 (20%)	2/5 (40%)	0/5 (0%)
Ljubljana	BP	0/5 (0%)	3/5 (60%)	0/5 (0%)	0/5 (0%)	0/5 (0%)	0/5 (0%)	0/5 (0%)	2/5 (40%)	0/5 (0%)	0/5 (0%)

**Table 4:** Number of tested bumblebee workers of six different species collected in Slovenia in 2017 and 2018.

Species/Year	2017	2018	Total
*Bombus terrestris/lucorum*	29	25	54
*Bombus lapidarious*	10	10	20
*Bombus sylvarum*	0	12	12
*Bombus pascuorum*	26	30	56
*Bombus hortorum*	0	3	3
*Bombus humilis*	0	2	2
All collected samples of *Bombus* spp.	65	82	147

## Data Availability

The data presented in this study are available on request from the corresponding author.

## References

[1] Potts S., Biesmeijer K., Bommarco R., Breeze T., Carvalheiro L., Franzen M., Gonzalez-Varo J.P., Holzschuh A., Kleijn D., Klein A.M. (2015). Status and Trends of European Pollinators. Key Findings of the STEP Project.

[2] Ollerton J., Winfree R., Tarrant S. (2011). How many flowering plants are pollinated by animals?. Oikos.

[3] Garibaldi L.A., Steffan-Dewenter I., Winfree R., Aizen M.A., Bommarco R., Cunningham S.A., Kremen C., Carvalheiro L.G., Harder L.D., Afik O. (2013). Wild Pollinators Enhance Fruit Set of Crops Regardless of Honey Bee Abundance. Science.

[4] Nieto A., Roberts S.P.M., Kemp J., Rasmont P., Kuhlmann M., Criado M.G., Biesmeijer J.C., Bogusch P., Dathe H.H., De la Rúa P. (2014). European Red List of Bees.

[5] Cameron S.A., Sadd B.M. (2020). Global Trends in Bumble Bee Health. Annu. Rev. Entomol..

[6] Glaum P., Simao M.C., Vaidya C., Fitch G., Iulinao B. (2017). Big city Bombus: Using natural history and land-use history to find significant envirnmental drivers in bumble-bee declines in urban development. R. Soc. Open Sci..

[7] Woodcock B.A., Isaac N.J.B., Bullock J.M., Roy D.B., Garthwaite D.G., Crowe A., Pywell R.F. (2016). Impacts of neonicotionoid use on long-term population changes in wild bees in England. Nat. Commun..

[8] Whitehorn P.R., O’Connor S., Wackers F.L., Goulson D. (2012). Neonicotinoid Pesticide Reduces Bumble Bee Colony Growth and Queen Production. Science.

[9] Rasmont P., Franzen M., Lecocq T., Harpke A., Roberts S.P.M., Biesmeijer J.C., Castro L., Cederberg B., Dvorak L., Fitzpatrick U. (2015). Climatic risk and distribution atlas of European bumblebees. BioRisk.

[10] Soroye P., Newbold T., Kerr J. (2020). Climate change contributes to widespread declines among bumble bees across continents. Science.

[11] Sokol R., Michalczyk M., Micholap P. (2018). Preliminary studies on the occurrence of honeybee pathogens in the national bumblebee population. Ann. Parasitol..

[12] Fürst M.A., McMahon D.P., Osborne J.L., Paxton R.J., Brown M.J.F. (2014). Disease associations between honeybees and bumblebees as threat to wild pollinators. Nature.

[13] Manley R., Boots M., Wilfert L. (2015). Emerging viral disease risk to pollinating insects: Ecological, evolutionary and anthropogenic factors. J. Appl. Ecol..

[14] McMahon D.P., Fürst M.A., Caspar J., Theodorou P., Brown M.J., Paxton R.J. (2015). A sting in the spit: Widespread cross-infection of multiple RNA viruses across wild and managed bees. J. Anim. Ecol..

[15] Meeus I., Brown M.J.F., Graaf D.C., Smagghe G. (2011). Effects of Invasive Parasites on Bumble Bee Declines. Conserv. Biol..

[16] Evison S.E.F., Roberts1 K.E., Laurenson L., Pietravalle S., Hui J., Biesmeijer J.C., Smith J.E., Budge G., Hughes W.O.H. (2012). Pervasiveness of Parasites in Pollinators. PLoS ONE.

[17] Goulson D., Hughes W.O.H. (2015). Mitigating the anthropogenic spread of bee parasites to protect wild pollinators. Biol. Conserv..

[18] Graystock P., Goulson D., Hughes W.O. (2015). Parasites in bloom: Flowers aid dispersal and transmission of pollinator parasites within and between bee species. Proc. R. Soc. B.

[19] Graystock P., Ng W.H., Parks K., Tripodi A.D., Muniz P.A., Fersch A.A., Myers C.R., McFrederick Q.S., McArt S.H. (2020). Dominant bee species and floral abundance drive parasite temporal dynamics in plant-pollinator communities. Nat. Ecol. Evol..

[20] Koch H., Brown M.J., Stevenson P.C. (2017). The role of disease in bee foraging ecology. Curr. Opin. Insect Sci..

[21] Adler L.S., Michaud K.M., Ellner S.P., McArt S.H., Stevenson P.C., Irwin R.E. (2018). Disease where you dine: Plant species and floral traits associated with pathogen transmission in bumble bees. Ecology.

[22] Colla S.R., Otterstatter M.C., Gegear R.J., Thomson J.D. (2006). Plight of the bumble bee: Pathogen spillover from commercial to wild populations. Biol. Conserv..

[23] Cameron S.A., Lim H.C., Lozier J.D., Duennes M.A., Thorp R. (2016). Test of the invasive pathogen hypothesis of bumble bee decline in North America. Proc. Natl. Acad. Sci. USA.

[24] Graystock P., Blane E.J., McFrederick Q.S., Goulson D., Hughes W.O.H. (2016). Do managed bees drive parasite spread and emergence in wild bees?. Int. J. Parasitol. Parasites Wildl..

[25] Graystock P., Goulson D., Hughes W.O.H. (2014). The relationship between managed bees and the prevalence of parasites in bumblebees. PeerJ.

[26] McNeil D.J., McCormick E., Heimann A.C., Kammerer M., Douglas M.R., Goslee S.C., Grozinger C.M., Hines H.M. (2020). Bumble bees in landscapes with abundant floral resources have lower pathogen loads. Sci. Rep..

[27] Toplak I., Šimenc L., Pislak Ocepek M., Bevk D. (2020). Determination of genetically identical strains of four honeybee viruses in bumblebee positive samples. Viruses.

[28] Gegear R.J., Otterstatter M.C., Thomson J.D. (2006). Bumble-bee foragers infected by a gut parasite have an impaired ability to utilize floral information. Proc. R. Soc. B.

[29] Fauser A., Sandrock C., Neumann P., Sadd B. (2017). Neonicotinoids override a parasite exposure impact on hibernation success of a key bumblebee pollinator. Ecol. Entomol..

[30] Brown M.J.F., Schmid-Hempel R., Schmid-Hempel P. (2003). Strong context-dependent virulence in a host-parasite system: Reconciling genetic evidence with theory. J. Anim. Ecol..

[31] Schwarz R.S., Bauchan G.R., Murphy C.A., Ravoet J., De Graaf D.C., Evans J.D. (2015). Characterization of two species of trypanosomatidae from the Honey Bee *Apis mellifera: Crithidia mellificae* Langridge and McGhee, and *Lotmaria passim* n. gen., n. sp.. J. Eukaryot. Microbiol..

[32] Stevanovic J., Schwarz R.S., Vejnovic B., Evans J.D., Irwin R.E., Glavinic U., Stanimirovic Z. (2016). Species-specific diagnostics of *Apis mellifera* trypanosomatids: A nine-year survey (2007–2015) for trypanosomatids and microsporidians in Serbian honey bees. J. Invertebr. Pathol..

[33] Van Der Steen J.J. (2008). Infection and transmission of *Nosema bombi* in *Bombus terrestris* colonies and its effect on hibernation, mating and colony founding. Apidologie.

[34] Otti O., Schmid-Hempel P. (2007). *Nosema bombi*: A pollinator parasite with detrimental fitness effects. J. Invertebr. Pathol..

[35] Otti O., Schmid-Hempel P. (2008). A field experiment on the effect of *Nosema bombi* in colonies of the bumblebee *Bombus terrestris*. Ecol. Entomol..

[36] Graystock P., Yates K., Darvill B., Goulson D., Hughes W.O. (2013). Emerging dangers: Deadly effects of an emergent parasite in a new pollinator host. J. Invertebr. Pathol..

[37] Gisder S., Horchler S., Pieper F., Schüler V., Šima P., Genersch E. (2020). Rapid Gastrointestinal Passage May Protect *Bombus terrestris* from ecoming a True Host for *Nosema ceranae*. Appl. Environ. Microbiol..

[38] Aizen M.A., Smith-Ramírez C., Morales C.L., Vieli L., Sáez A., Barahona-Segovia R.M., Arbetman M.P., Montalva J., Garibaldi L.A., Inouye D.W. (2019). Coordinated species importation policies are needed to reduce serious invasions globally: The case of alien bumblebees in South America. J. Appl. Ecol..

[39] Rutrecht S.T., Brown M.J. (2008). The life-history impact and implications of multiple parasites for bumble bee queens. Int. J. Parasitol..

[40] Graystock P., Meeus I., Smagghe G., Goulson D., Hughes W.O. (2016). The effects of single and mixed infections of *Apicystis bombi* and deformed wing virus in *Bombus terrestris*. Parasitology.

[41] Peng W., Li J., Boncristiani H., Strange J.P., Hamilton M., Chen Y. (2011). Host range expansion of honey bee black queen cell virus in the bumble bee, *Bombus huntii*. Apidologie.

[42] Meeus I., de Miranda J.R., de Graaf D.C., Wäckers F., Smagghe G. (2014). Effect of oral infection with Kashmir bee virus and Israeli acute paralysis virus on bumblebee (*Bombus terrestris*) reproductive success. J. Invertebr. Pathol..

[43] Genersch E., Yue C., Fries I., de Miranda J.R. (2006). Detection of Deformed wing virus, a honey bee viral pathogen, in bumble bees (Bombus terrestris and Bombus pascuorum) with wing deformities. J. Invertebr. Pathol..

[44] Cilia G., Zavatta L., Ranalli R., Nanetti A., Bortolotti L. (2021). Replicative Deformed Wing Virus Found in the Head of Adults from Symptomatic Commercial Bumblebee (*Bombus terrestris*) Colonies. Vet. Sci..

[45] Pascall D.J., Tinsley M.C., Obbard D.J., Wilfert L. (2019). Host evolutionary history predicts virus prevalence across bumblebee species. bioRxiv.

[46] Gogala A. (2014). Čebele Slovenije.

[47] Toplak I., Jamnikar-Ciglenečki U., Aronstein K., Gregorc A. (2013). Chronic bee paralysis virus and Nosema ceranae experimental co-infection of winter honey bee workers (*Apis mellifera L*.). Viruses.

[48] Toplak I., Rihtarič D., Jamnikar Ciglenečki U., Hostnik P., Jenčič V., Barlič-Maganja D. (2012). Detection of six honeybee viruses in clinically affected colonies of Carniolan gray bee (*Appis mellifera carnica*). Slov. Vet. Res..

[49] Fries I., Chauzat M.P., Chen Y.P., Doublet V., Genersch E., Gisder S., Higes M., McMahon D.P., Martín-Hernández R., Natsopoulou M. (2013). Standard methods for Nosema research. J. Apic. Res..

[50] Meeus I., de Graaf D.C., Jans K., Smagghe G. (2010). Multiplex PCR detection of slowly-evolving trypanosomatids and neogregarines in bumblebees using broad-range primers. J. Appl. Microbiol..

[51] Xu G., Palmer-Young E., Skyrm K., Daly T., Sylvia M., Averill A., Rich S. (2018). Triplex real-time PCR for detection of *Crithidia mellificae* and *Lotmaria passim* in honey bees. Parasitol. Res..

